# Nutritional Value of Glycerin for Pigs Fed a Mixture or an On-Top Diet

**DOI:** 10.3390/ani15101387

**Published:** 2025-05-11

**Authors:** Rafaeli Gonçalves Leite, Alexandre de Oliveira Teixeira, Charles Kiefer, Ana Paula Silva Ton, Maicon Sbardella, Claudson Oliveira Brito, Leonardo Willian de Freitas, Anderson Corassa

**Affiliations:** 1 Agrarian and Environmental Sciences Institute, Federal University of Mato Grosso, Sinop 78550-000, Brazil; rafaelileite.rgl@gmail.com (R.G.L.); ana.ton@ufmt.br (A.P.S.T.); maicon.sbardella@ufmt.br (M.S.); 2 Department of Animal Science, Federal University of São João del-Rei, São João del-Rei 36307-352, Brazil; alexandre_teixeira@ufsj.edu.br; 3 Veterinary and Animal Science Department, Federal University of Mato Grosso do Sul, Campo Grande 79070-900, Brazil; charles.kiefer@ufms.br; 4 Animal Science Department, Federal University of Sergipe, São Cristovão 40035-530, Brazil; claudson@academico.ufs.br

**Keywords:** alternative food, biodiesel co-product, digestibility, glycerol, metabolizable energy

## Abstract

Glycerin has a similar energy value to corn and can partially replace it. However, there is a need to find the ideal level for using glycerin in pig feed without negatively interfering with performance parameters. The objective of this study was to determine the digestibility of glycerin through a mixture (MIX) or inserted on top of feed (ONTOP) using chromium indicator (Cr) and total collection (TC) methods. Ten barrows weighed 42.63 ± 4.23 kg in a 2 × 2 + 1 factorial scheme, with two forms of inclusion of the test ingredient (MIX or ONTOP), two digestibility methods (Cr or TC), and a basal diet (BD). The experimental design was completely randomized, and the evaluation period was used as split-plots, with two repetitions per period, totaling four repetitions per diet. The DE and ME values for glycerin were 3443 and 3356 kcal/kg (by the TC method) and 3411 and 3293 kcal/kg (by the Cr method), respectively (*p* < 0.05). There was no difference in MIX or ONTOP (*p* > 0.05), except for the digestibility coefficient (DC) of ethereal extract (*p* < 0.05). The DC and energy values obtained by Cr were lower than those obtained by TC. The inclusion of the test ingredients in the MIX and ONTOP forms did not present any difference, whereas Cr depreciated the nutritional value of glycerin for pigs.

## Simple Summary

Glycerin, a by-product of biodiesel production, can be used as a high-potential energy source for pig feed, as it has similar energy values to corn and wheat. Glycerin can be considered an alternative ingredient to replace these traditional foods in pig feed. The aim of this study was to analyze the digestibility of glycerin when mixed directly into the feed (MIX) or applied on top (ONTOP) using two methods, i.e., chromium indicator (Cr) and total collection (TC). Ten barrows with an average weight of 42.63 kg were used. The experiment was conducted in a 2 × 2 + 1 format, with two forms of glycerin inclusion (MIX or ONTOP), two methods of measuring digestibility (Cr or TC), and a basal diet (BD). The experiment was in a completely randomized design, and the evaluation period was used as split-plots, with two repetitions per period, totaling four repetitions per diet. The DE and ME values for glycerin were 3443 and 3356 kcal/kg (by the CT method) and 3411 and 3293 kcal/kg (by the Cr method), respectively. There was no significant difference between the inclusion forms (MIX or ONTOP), except for fat digestibility. Furthermore, the values obtained by the Cr method were lower than those obtained by the TC method. It is concluded that the form of inclusion of glycerin in the diet had no significant impact, but the Cr method underestimated the nutritional value of glycerin for pigs.

## 1. Introduction

The increasing demand for transportation fuels, coupled with decreasing crude oil reserves and a growing awareness of climate change, has increased global interest in biodiesel [1]. Biodiesel is produced by the transesterification of vegetable oils or animal fats with an alcohol present as a catalyst. This reaction yields a considerable amount of crude glycerin as a byproduct, approximately 10% by weight of the biodiesel produced [2].

Global biodiesel production reached nearly 59 billion liters in 2022, with the main uses being palm oil, soybean oil, rapeseed oil, and animal fats [3]. The availability of byproducts such as glycerin jumped from 290K M^3^ in 2013 to 552K M^3^ in 2022, with soybean oil being the raw material that accounted for 66% of the total [4]. The continued growth in biofuel production has led to a search for alternative value-added applications for the use of glycerin. The surplus of glycerin production and increased cost of feedstuffs have increased the emphasis on evaluating its nutritive value for animal feed [5].

Glycerin has a similar energy value to corn and can partially replace it [6]. However, there is a need to find the ideal level for using glycerin in pig feed without negatively interfering with performance parameters [7].

The utilization of glycerin in pig diets has increased due to its great advantages, but the success of its use is related to the knowledge of its characteristics and nutritional value. Studies using glycerin for pigs have been conducted, and glycerin has been shown to be a good digestible ingredient and source of energy [7,8,9]. A consensus has not been reached, as the metabolizable energy (ME) value ranges from 2531 kcal/kg [10] to 5509 kcal/kg [11].

In many countries, combining a food compound, such as feed, with water or industrial liquid byproducts has gained popularity as a feeding method. Its effects on digestibility and weight gain have been demonstrated for Sol et al. [12]. However, the use of glycerin in diets can lead to operational difficulties in the feed manufacturing and supply process, with effects on animal performance. Reports of lump formation and poor fluidity [13] and low-quality pellets [14,15] have reflected 5% maximum inclusions for pellet durability [16], improvement in feed fluidity [17], and increased pelleting efficiency [18], thus limiting the use of higher glycerin inclusions. Thus, the inclusion of glycerin just before the start of feeding (similar to liquid diets), called “on top”, allows for greater inclusions of glycerin without compromising the processing of the diets.

The method used to determine the energy content may influence the nutritional value of glycerin. Either the total collection method (TC) or the chromic oxide index method (Cr) can be used. TC is one of the most commonly used methods for determining the digestibility of nutrients as well as the values of digestible energy (DE) and ME, but obtaining representative samples without contamination is problematic [19,20]. The index method requires including a certain concentration of indigestible compound in the diet and allows partial sampling but requires a precise chemical analysis of the indigestible markers [20,21,22].

Therefore, the objective was to determine the digestibility of glycerin by inclusion in the feed mixture and addition on top by the TC and Cr methods.

## 2. Materials and Methods

The experiment was carried out in the experimental warehouse of the Nonruminant Nutrition Research sector of the Institute of Agricultural and Environmental Sciences, Federal University of Mato Grosso (Sinop, Mato Grosso, Brazil, latitude −11°86′26″ and longitude −55°48′49″). The research complied with the ethical principles of animal experimentation adopted by the National Council for Animal Experimentation Control and previously approved by the Ethics Committee in the Use of Farming Animals at the Federal University of Mato Grosso (protocol number 23108.700673/14-4).

A total of ten genetically homogeneous barrows (42.63 ± 4.23 kg) were distributed individually into metabolic study cages in a 2 × 2 + 1 factorial arrangement, with two forms of inclusion of the test ingredient (glycerin mixed with the feed beforehand (MIX) and glycerin included immediately before the start of feeding (ON TOP)), two digestibility methods (total collection and chromium indicator), and a basal diet (Table 1). The experimental design was completely randomized, and the evaluation period was used as split-plots, with two repetitions per period. Each pig was considered an experimental unit, with four replicates per treatment.

The basal diet (BD) was produced from corn and soybean meal [23] (Table 2). To evaluate the forms of inclusion of the test ingredients, the isometric substitution of 10% BD by glycerin and a new mixture (MIX) was performed [19], and the 10% isometric substitution of BD for glycerin was carried out immediately prior to delivery to the animals, called “on top” (ONTOP) (Table 3).

In all diets, 0.5% chromic oxide (Cr_2_O_3_) was added [19], thus allowing the evaluation of digestibility through the chromium indicator (Cr) and total collection (TC) methods. BD was 90.00, 89.76, 4.09, 10.24, and 3943 kcal/kg, and MIX and ONTOP were 88.00, 91.07, 6.72, 9.69, and 3980 kcal/kg DM, OM, EE, ash, and GE, respectively.

The glycerin used here was obtained from the production of biodiesel, with soybean oil as the raw material, with 0.49% humidity, 86.5% glycerol, 0.05% methanol, and 0.30% total fatty acids, according to the technical report provided by the manufacturer.

The experimental period was eight days, i.e., three days of adaptation of the treatments to the cages and diets and five days of feces and urine collection [19]. During the adaptation period, the diet was supplied ad libitum, and orts were recorded for a later calculation of intake on the basis of metabolic weight (LW^0.75^). To prevent losses and facilitate intake, diets were weighed and moistened at a 1:1 ratio and supplied twice daily (07:30 and 17:00) [24].

Feces and urine were collected once daily, weighed and homogenized, and then, 200 g/kg samples were stored in a freezer (−10 °C). The urine was filtered as it was excreted using a filter tissue placed in a funnel beneath the urine collection box and then collected in plastic buckets containing 10 mL of 6 N HCl [25].

At the end of the collection period, the diet and feces samples were thawed, weighed, homogenized, and dried in a forced-air oven at 55 °C for 72 h for analyses of dry matter (DM, method 934.01) [26], crude protein (CP, method 2001.11) [26], ether extract (EE, method 945.38) [26], and ash (method 923.03) [26], and gross energy (GE) values of the feces, urine, diets, and coproducts were determined using a bomb calorimeter (Parr 6400 calorimeter, Parr Instruments Co., Moline, IL, USA) (Table 4). The organic matter (OM) content was determined as the difference between the DM and ash contents [19]. Analyses of the chromium content in the diets and feces were performed via atomic absorption spectrophotometry [7]. The urine samples were thawed and homogenized for the determination of total nitrogen and gross energy. The digestibility coefficients (DCs) of DM, ash, OM, EE, energy, DE, ME, and the corrections for the nitrogen content (DEn and MEn) were determined [19].

The animals were weighed at the beginning and end of each period, and the feed intake was recorded to calculate the daily feed intake (DFI), daily weight gain (DWG), and feed conversion (FC) of each experimental unit.

The digestibility and energy data were performed according to the following model (1):

(1) Yij=μ+Ii+Mj+Ii×Mj+eij

where *Yij* = observation of the effect of the form of inclusion *i* by the digestibility assessment method *j*; *μ* = overall mean; *Ii* = effect of the form of inclusion *i* (*i* = mixture or on top); *Mj* = effect of the digestibility method k (k = total collection and marker); *Ii* × *Mj* = effect of the interaction between the form of inclusion *i* and the digestibility method *j*; and *eij* = random error associated with each observation.

The data were subjected to ANOVA using the GLM (general linear model) procedure of SAS software (Statistical Analysis System, version 6.0), considering the 5% probability by Tukey test for performance and the F test for DC and energy values.

## 3. Results

Compared with those fed diets containing glycerin, the animals fed with a basic diet presented a greater DFI (*p* < 0.05), but there was no difference in DWG or FC among the treatments (*p* > 0.05) (Table 4).

The DM, MM, and OM DC of glycerin were not influenced by the inclusion form of the test ingredient in the diet (*p* > 0.05), but ONTOP inclusion generated a greater EE DC (*p* < 0.05) (Table 4). Compared with those of the TC samples, the glycerin DC samples presented lower values when generated by the Cr method compared to TC (Table 5). These findings imply that the top form with a 10% inclusion level can be used without detriment to the nutritional value of glycerin for pigs if it is more feasible in terms of logistics.

The values of DE, DEn, ME, MEn, ME:DE, and MEn:DEn of glycerin were not influenced by the inclusion form (*p* > 0.05); however, for all these parameters, the values of the Cr method were smaller than those of TC (*p* < 0.05) (Table 6).

## 4. Discussion

The lower feed intake of the animals fed diets containing glycerin may be related to the characteristics of the test ingredients. Although glycerin has a sweet taste [16], this ingredient may influence feed intake due to impurities such as sodium, methanol, and mineral matter, suggesting adjustments in diet composition, such as electrolyte balance [9,18,27].

In this sense, Hansen et al. [28] reported a decrease in the DFI of the animals as the glycerin level (0–16%) in the first week of supply increased, suggesting that the period of adaptation of the animals to the new ingredient was necessary. However, other studies [7,9] reported no change in the DFI when glycerin up to 15% was included in the diet.

The form of inclusion of the test ingredient did not influence the DCs except for EE. These findings imply that the top form with a 10% inclusion level can be used without detriment to the nutritional value of glycerin for pigs if it is more feasible in terms of logistics. Inclusions of up to 15% glycerin in diets for weaned piglets did not affect DM, CP, or GE digestibility despite a linear increase in urinary production [29]. Evaluating inclusions up to 10%, Martínez-Miró et al. [1] reported no effect on the apparent total tract digestibility of DM, OM, and CP or on the levels of IGF-1, serum protein, insulin, glucose, and albumin in finishing Iberian crossbred pigs. However, the potential of glycerol to promote the proliferation of advantageous bacteria at the gut level was investigated by Wei et al. [30]. They discovered that by altering the makeup of the fecal microbiota and metabolites, glycerol, when paired with other nutrients, may be a useful method of enhancing muscle redness in pigs.

Although glycerin can be used as a humectant and sweetener, providing flavor and color to food [31], previous studies have shown worse pellet quality [15], flowability in feeders [13], and animal performance [14] when glycerin inclusion is greater than 10%, probably due to difficulties in dosing and homogenization during mixing.

The mixing process is indispensable in feed manufacturing to support all nutrients in pigs. The use of liquid ingredients or mixing with water can interfere with the mixing, consumption, and digestibility of animal diets. Thus, glycerin, which is a liquid ingredient, can improve the mixture when it is present in small proportions. In accordance with the findings of Brooks [32], the process of combining water with dry feed and subsequently providing it to pigs after a brief interval ensures a more consistent diet. This, in turn, accelerates the hydration process, particularly when the feed is finely ground, thereby supporting the optimal function of both digestive and in-feed enzymes.

The use of glycerin as a liquid ingredient, whether in a mixture or on top, provides new insights into enhancing the nutritional use of diets with different physical presentations. The organic matter digestibility and gross energy digestibility of pigs fed water-to-feed ratios of 2.1:1 and 2.7:1 were greater than those of pigs not fed water [12].

All the glycerin DCs presented lower values when generated by the Cr method than when generated via the TC method (*p* < 0.05) (Table 5). The DC and glycerin energy values obtained by Cr underestimated the values in relation to TC. This finding may be related to the nontotal indigestibility, recovery of chromium in the feces, quality of the analysis [21], quality of the mixture, and variations in the diets [33]. Studies that evaluated glycerin [7] and dried distiller grains with soluble [34] also reported lower values of energy and DC determined with Cr than with TC. The results of this study are similar to those of Liu [35], who reported that the apparent total tract digestibility (ATTD) values of GE, DM, OM, EE, CP, and neutral detergent fiber (NDF) were lower when the Cr method was used than when the TC method was used because of the recovery of markers in feces, necessitating further investigations.

Nevertheless, a study by Huang et al. [36] revealed that the DE, ME, and ATTD of GE, ash, NDF, and acid detergent fiber of experimental diets determined using Cr markers were greater than those determined using the TC method. In this context, Prawirodigdo et al. [37] reported a significant correlation (R^2^ = 0.86, 0.88, 0.74) for the ATTD of nitrogen, OM, and DM in experimental diets between Cr_2_O_3_, an indigestible marker, and total fecal collection. However, the authors reported that some results were smaller or larger depending on the basal diet and ingredient tested, so it appears that the amount and type of protein source can influence the reliability of the Cr_2_O_3_ marker, especially for the ATTD of N.

The DC and glycerin energy values obtained with Cr were lower than those obtained with the total collection method. Kavanagh et al. [38] reported that markers in feed could be lost at the mixing stage and sampling of feed, resulting in variations in marker analysis. Agudelo et al. [39] reported that the values of digestibility for DM and energy were lower for chromium oxide than for total collection and that the indicator method was less able to detect differences in ingredients with highly concentrated nutrients. In this sense, the results of Wang et al. [40] also revealed that the apparent digestibility of energy determined by TM was greater than that determined by Cr_2_O_3_ or TiO_2_. The results of Jang et al. [41] indicated that Cr results in lower digestibility values, when compared to the TC method, do not provide the same treatment difference as the TC digestibility for energy and nutrients that are not highly impacted by dietary treatment. In addition, the TC data seemed to have a lower coefficient of variation than the indicator method data for many components. Therefore, total collection has been considered the “gold standard”, as all voided nutrients are supposedly collected.

According to a recent review published by Zhang and Adeola [21], the markers should be (1) totally indigestible and nonabsorbable, (2) nontoxic to the digestive tract, (3) pass through the digestive tract at a relatively uniform rate with digesta, and (4) easy to analyze.

The energy value of glycerin is the main highlight of this ingredient. When values close to those of corn are presented, it becomes a potential substitute in diets for pigs. The glycerin values of GE (5397 kcal/kg), DE (3443, 3411 kcal/kg), and ME (3356, 3293 kcal/kg) determined in this study were close to 3387 and 3270 kcal/kg [7] but were lower than 5240, 5070, and 4556 kcal/kg [23] and 6500, 5839, and 5509 kcal/kg [11].

The difference in the DE and ME for glycerin in this study and others is related to the raw material, biodiesel process, glycerol, and lower fatty acid contents [42]. Glycerol from glycerin is absorbed by passive diffusion and metabolized into glucose via phosphorylation to glycerol-3-phosphate by glycerin kinase [43], with excess being excreted in the urine [44] but no saturation up to 15% glycerin in the diet [45].

## 5. Conclusions

The glycerin had 3443 and 3411 kcal/kg of digestible energy and 3356 and 3293 kcal/kg of metabolizable energy for pigs, as determined by the total collection and indicator methods, respectively.

The inclusion of glycerin in the mixture or on top did not affect the digestibility coefficients or energy for pigs, but the values were lower when the addition of chromium oxide was used than when the total collection method was used.

Glycerin can be considered an alternative ingredient in replacing energy ingredients, and the incorporation of glycerin in mashed feeds can bring benefits such as greater energy density, better palatability, and reduced powderiness, while the inclusion “on top” allows us to make larger inclusions without harming the manufacturing process.

## Figures and Tables

**Table 1:** Experimental layout for trial.

A 2 × 2 + 1 Factorial Arrangement		Digestibility Methods	
forms of inclusion of the glycerin	MIX	Cr	TC
	ON TOP	Cr	TC
(+1) basal diet			

MIX: glycerin mixed with the feed beforehand; ON TOP: glycerin included immediately before the start of feeding; Cr: chromium indicator method; TC: total collection method (urine and feces).

**Table 2:** Composition and calculated nutritional values of the reference diet (as-fed basis).

Ingredient (%)	Reference Diet
Corn	60.48
Soybean meal	30.26
Rice bran	3.00
Soy oil	1.89
Calcitic limestone	0.52
Dicalcium phosphate	1.75
Vitamin–mineral mix ^1^	1.00
Common salt	0.46
L-lysine	0.15
Chromium oxide	0.50
Total	100
Calculated nutrient content, %	
Metabolizable energy, kcal/kg	3230
Crude protein	18.99
Calcium	0.72
Available phosphorus	0.36
Sodium	0.20
Digestible lysine	1.01

^1^ Composition of the supplement per kg of diet: vitamin A (13750 UI), vitamin B1 (2 mg), vitamin B2 (1,25 mg), vitamin B6 (4 mg), vitamin B12 (4.5 mcg), vitamin D3 (3000 UI), vitamin E (75 UI), vitamin K3 (6.25 mg), nicotinic acid (50 mg), pantothenic acid (30 mg), folic acid (0.625 mg), cobalt (1.25 mg), copper (25 mg), iron (150 mg), zinc (200 mg), manganese (75 mg), selenium (0.7 mg), iodine (2 mg), choline (250 mg), and biotin (25 mcg).

**Table 3:** Chemical composition of diets (% in dry matter).

Item	BD	MIX	ONTOP
Dry matter (%)	89.99	88.0	88.0
Organic matter (%)	89.76	91.07	91.07
Ash (%)	10.24	9.69	9.69
Ether extract (%)	4.09	6.72	6.72
Gross energy (kcal/kg)	3943	3980	3980

**Table 4:** Daily weight gain (DWG), daily feed intake (DFI), and feed conversion (FC) of pigs during the digestibility trial fed base diet (BD), with the inclusion of glycerin on mix (MIX) and the inclusion of glycerin on top (ONTOP).

Item	BD	MIX	ON TOP	*p*-Value	SEM ^1^
DFI (g/day)	1886 ^a^	1764 ^b^	1800 ^b^	0.0231	42.48
DWG (g/day)	621	517	596	0.3706	56.56
FC (kg/kg)	3.15	3.65	3.03	0.4487	0.30

^1^ Standard error of the mean. Means followed by different letters on the line differ statistically at 5% probability according to the Tukey test.

**Table 5:** Digestibility coefficients of the chemical composition of glycerin for pigs with different glycerin levels determined by inclusion of glycerin in the mix (MIX), the inclusion of glycerin on top (ONTOP), and total collection (TC) and chromium marker (Cr) digestibility methodologies.

DC ^1^ (%)	Inclusion		Method		*p*-Value			SEM ^6^
	MIX	ONTOP	TC	Cr	Inclusion	Method	I × M ^5^	
DM ^2^	91.57	91.59	87.13 ^a^	86.02 ^b^	0.8185	<0.0001	0.7396	0.09
Ash	84.66	84.74	94.56 ^a^	74.84 ^b^	0.5279	<0.0001	0.4170	0.10
OM ^3^	87.97	87.99	88.35 ^a^	87.61 ^b^	0.8213	0.0002	0.8174	0.09
EE ^4^	76.47 ^b^	76.99 ^a^	77.43 ^a^	76.03 ^b^	0.0441	<0.0001	0.9913	0.11

^1^ Digestibility coefficient; ^2^ dry matter; ^3^ organic matter; ^4^ ether extract; ^5^ inclusion form of glycerin × digestibility methodology interaction; and ^6^ standard error of the mean. Means followed by different letters on the line differ statistically at 5% probability according to the F test.

**Table 6:** Energy-related variables of glycerin were determined via glycerin on mix (MIX), glycerin on top (ONTOP), and total collection (TC) and chromium marker (Cr) digestibility methodologies.

Energy (kcal/kg)	Inclusion		Method		*p* Value			SEM ^6^
	MIX	ONTOP	TC	Cr	Inclusion	Method	I × M ^5^	
DE ^1^	3426	3428	3443 ^a^	3411 ^b^	0.7045	0.0002	0.8337	3.43
DEn ^2^	3413	3414	3429 ^a^	3398 ^b^	0.7092	0.0002	0.8334	4.10
ME ^3^	3322	3326	3356 ^a^	3293 ^b^	0.4030	<0.0001	0.4224	3.32
MEn ^4^	3310	3314	3344 ^a^	3279 ^b^	0.4157	<0.0001	0.4301	4.80
ME:DE	0.9695	0.9702	0.9746 ^a^	0.9652 ^b^	0.3109	<0.0001	0.1745	0.01
MEn:DEn	0.9698	0.9704	0.9751 ^a^	0.9651 ^b^	0.3385	<0.0001	0.1791	0.01

^1^ Digestible energy; ^2^ digestible energy corrected for nitrogen balance; ^3^ metabolizable energy; ^4^ MEn metabolizable energy corrected for nitrogen balance; ^5^ inclusion form of glycerin × digestibility methodology interaction; and ^6^ standard error of the mean. Means followed by different letters on the line differ statistically at 5% probability according to the F test.

## Data Availability

The raw data supporting the conclusions of this article will be made available by the authors without undue reservation.

## Abbreviations

The following abbreviations are used in this manuscript:

MIX	mixture
ONTOP	on top of feed
Cr	chromium indicator
TC	total collection
BD	basal diet
DC	digestibility coefficient
DM	dry matter
OM	organic matter
EE	ethereal extract
DE	digestible energy
ME	metabolizable energy
ATTP	apparent total tract digestibility
DFI	daily feed intake
DWG	daily weight gain
FC	feed conversion
NDF	neutral detergent fiber
